# Low‐Dose Nivolumab in Various Cancers: A Real‐World Exploratory Analysis in Resource‐Constrained Settings

**DOI:** 10.1002/cnr2.70713

**Published:** 2026-10-19

**Authors:** Subhas Pandit, Simit Sapkota, Abish Adhikari, Jeebana Bhandari, Sunil Shrestha, Rajani Jha, Gerim Prasai, Anjani Kr. Jha

**Affiliations:** ^1^ Department of Clinical Oncology Kathmandu Cancer Center Bhaktapur Nepal; ^2^ Department of Research and Academics Kathmandu Cancer Center Bhaktapur Nepal; ^3^ Department of Medical Oncology Kathmandu Cancer Center Bhaktapur Nepal

**Keywords:** financial toxicity, immune checkpoint inhibitors, immunotherapy, low‐dose nivolumab, PD‐1/PD‐L1 interaction

## Abstract

**Background:**

Immune checkpoint inhibitors (ICIs), including nivolumab, have significantly improved cancer care. However, due to high costs, there is a significant financial barrier to their widespread access. Emerging evidence shows biological plausibility as well as efficacy of low‐dose nivolumab across different cancers.

**Aims:**

This exploratory study aims to investigate the clinical outcomes of low‐dose nivolumab in cancer treatment to explore the potential to improve its access by reducing costs, especially in resource‐limited settings.

**Methods and Results:**

Data on patients with various malignancies treated with fixed 20 or 40 mg of nivolumab every 3 weeks were retrospectively analyzed. Response to the treatment, including the best objective response rate, best disease control rate, and adverse events, was reported. The Kaplan–Meier method was used to determine progression‐free survival and overall survival. The patient cohort of 31 patients with various tumors received a median of 9 cycles (range, 2–20) of low‐dose nivolumab therapy for 5.7 months (range, 0.7–17.1). Median follow‐up was 8.6 months (95% CI, 7.1–12.9). Disease control was achieved in 21/31 (67.7%) patients, with the best objective response rate of 51.6%. The median progression‐free survival was 8.8 months (95% CI, 6.6‐not reached) and overall survival was 14.8 months (95% CI, 11.1‐not reached). At the end of the study period, 3/31 patients had a radiologic complete response, 9/31 had a partial response, 4/31 had stable disease, and 6/31 had progressive disease, and 3/31 were not evaluable; 6 patients died during this period. The responses achieved were sustainable for a median of 4 months (range, 0.8–8.2). Only 5 patients developed adverse events of grade 2 or 3.

**Conclusions:**

Low‐dose nivolumab shows preliminary efficacy in resource‐constrained settings. Further research should explore optimal dosing and scheduling for low‐dose immunotherapy and evaluate its efficacy and safety through larger, homogeneous studies.

## 1 Introduction

Cancer immunotherapy targeting programmed death–1 receptor and its ligand (PD‐1/PD‐L1) causes durable antitumor immunity [1]. Various checkpoint inhibitors targeting the PD‐1/PD‐L1 pathway, including nivolumab and pembrolizumab, have been approved for diverse cancers such as melanoma, lung cancer, and renal cell carcinoma [2]. However, the high costs of these innovative immunotherapies remain a significant barrier to their widespread access, particularly in resource‐limited settings, where most patients cannot afford these life‐saving treatments.

Recently, the necessity to continue treatment with approved, high doses of nivolumab is being challenged by emerging evidence. Phase 1 trials failed to show a maximum tolerated dose (MTD) and a dose–response relationship across a wide dose range of 0.1 to 10 mg/kg [3]. Similarly, a randomized Phase 2 study demonstrated a similar dose–response for 0.3, 2, and 10 mg/kg nivolumab in metastatic renal cell carcinoma [4]. These findings suggest the therapeutic efficacy of nivolumab at lower than approved doses, addressing financial toxicity and increasing access.

Immunotherapy is out of reach for more than 90% of patients in low and middle‐income countries (LMICs) due to high cost. The approved dose of nivolumab is 3 mg/kg or a fixed dose of 240 mg every 2 weeks or 480 mg every 4 weeks. Clinicians in resource‐limited settings are often forced to lower the doses of nivolumab empirically or abandon the treatment entirely. Only limited evidence suggests that lower, more affordable doses may also confer therapeutic efficacy comparable to the standard dose. To address this research gap, we investigated the clinical outcomes of ultralow‐dose (20 and 40 mg) nivolumab in diverse cancer patients. This study aims to provide real‐world data for dose optimization of nivolumab in resource‐limited settings, where balancing efficacy and affordability could enhance access to immunotherapy.

## 2 Materials and Methods

### 2.1 Patients and Treatment

This retrospective analysis included cancer patients who received low‐dose nivolumab at a single cancer center between March 2022 and March 2024. Low‐dose nivolumab (20 or 40 mg) was administered to patients with advanced‐stage cancer who showed limited response to conventional treatments. The low‐dose regimen was selected due to the patients' inability to afford the standard dose. Patients were explicitly informed about the limited evidence for the off‐label use of nivolumab. Doses were chosen based on prior studies demonstrating positive outcomes with reduced‐dose nivolumab [5, 6]. The choice between 20 and 40 mg doses depended on physician's preference and the patient's financial situation. Vial sharing was implemented since nivolumab was available in 40 mg vials at the hospital pharmacy. A few patients underwent PD‐L1 testing. Treatment continued until disease progression, unacceptable toxicity, or financial hardship prevented further therapy. Adverse events documented in routine medical records were retrospectively abstracted and graded according to the National Cancer Institute Common Terminology Criteria for Adverse Events (CTCAE) version 5.0. Because Grade 1 adverse events were not systematically documented during routine outpatient visits, the safety analysis was limited to reliably recorded adverse events. While iRECIST is considered an immune‐adapted response framework in ICIs, treatment response was evaluated using available radiological imaging (ultrasonography, CT scan, MRI, or PET‐CT). Ultrasound was performed in cases of nontarget and nonmeasurable diseases, for example, pleural effusion. Responses in patients with solid tumors were assessed according to Response Evaluation Criteria in Solid Tumors (RECIST 1.1) [7]. The response of the patient with Hodgkin lymphoma was assessed according to the Lugano classification. The Hodgkin lymphoma patient was included in the overall cohort response analysis but was not assessed using RECIST 1.1. Progression was defined only when an unequivocal increase or new appearance consistent with malignant progression was observed. Patients who received only one dose of nivolumab or whose data were incomplete were excluded from the analysis. Data on demographics, disease history, immunotherapy regimen (including dose and compliance), and adverse events were retrospectively collected from patient medical records.

### 2.2 Ethical Approval and Informed Consent

This study was approved by the Nepal Health Research Council (approval no. 674/2023) and conducted in accordance with the Declaration of Helsinki. All patients provided written informed consent for the off‐label use of low‐dose nivolumab, acknowledging the limited evidence for these doses and that the treatment was chosen due to financial constraints.

### 2.3 Statistical Analysis

Continuous variables were expressed as the median and categorical variables as counts and percentages. The median duration of response was determined for patients exhibiting either complete response (CR) or partial response (PR), starting from the onset of response to either subsequent disease progression or the conclusion of the study period. Median follow‐up duration was calculated using the reverse Kaplan–Meier method, censoring the dead patients at their month of death [8].

The overall response rate (ORR) was calculated as the proportion of all included patients who achieved a complete or partial response according to the disease‐appropriate response criteria.

The disease control rate (DCR) was defined as the ratio of patients with any response or stable disease to the entire cohort. Two‐sided 95% confidence intervals for ORR and DCR were calculated using the Clopper–Pearson exact binomial method. Survival analyses were performed using the Kaplan–Meier method. Progression‐free survival (PFS) was measured from the date of first nivolumab administration until either progression or death due to any cause. Overall survival (OS) was measured from the date of first nivolumab administration until either death due to any cause or the last follow‐up date. All statistical analyses were performed using R Statistical Software (v4.3.2; R Core Team) [9].

## 3 Results

### 3.1 Patients

From March 2022 through March 2024, a total of 31 patients received low‐dose nivolumab. The median age of the patients was 51 years (range, 28–87). The demographic and clinical characteristics of the patients are summarized in Table 1. Around 26.8% of patients received nivolumab as first‐line therapy, while other patients received it as a subsequent line of therapy after prior chemotherapy regimens. Five patients received cytotoxic chemotherapy (Capecitabine/Oxaliplatin‐1, Paclitaxel/Carboplatin‐3, Irinotecan‐1) in combination with nivolumab, while others received nivolumab as a single agent.

**TABLE 1:** Baseline characteristics of patients.

	Number (*n* = 31)	Percentage
Sex		
Male	17	54.8
Female	14	45.2
ECOG PS		
ECOG 0	11	35.5
ECOG 1	12	38.7
ECOG 2	5	16.1
ECOG 3	3	9.7
PD‐L1 status		
Known	6	19.4
Unknown	25	80.6
Lines of prior systemic therapy		
None	8	25.8
One	16	51.6
Two	5	16.1
Three or more	2	6.5
System		
Gastrointestinal	11	35.5
Lungs	6	19.4
Head & Neck	9	29.0
Gynecologic	3	9.7
Others (Skin/Hodgkin's lymphoma^a^)	2	6.5

^a^ Hodgkin lymphoma response was assessed using the Lugano classification.

### 3.2 Treatment Outcomes

Responses to low‐dose nivolumab are depicted in Table 2. Patients received a median of 9 cycles (range, 2–20) of nivolumab for a median of 5.7 (range, 0.7–17.1) months. The most frequently administered dose was 20 mg in 64.5% of patients, followed by 40 mg in 35.5% of patients.

**TABLE 2:** Response to low‐dose nivolumab.

	Number (*n* = 31)	Percentage
Dose of nivolumab		
20 mg	20	64.5
40 mg	11	35.5
Best response		
Complete response (CR)	4	12.9
Partial response (PR)	12	38.7
Stable disease (SD)	5	16.1
Progressive disease (PD)	7	22.6
Not evaluated	3	9.7
Patient's status at the end of the study period		
Alive without disease	3	9.7
Alive with disease	22	71.0
Dead	6	19.4
Concomitant chemotherapy	5^a^	16.1
Subsequent therapy		
No further treatment	27	87.1
Chemotherapy	3	9.7
Targeted therapy	1	3.2

^a^ One complete responder, two partial responders, and two patients with progressive diseases received low‐dose nivolumab with chemotherapy.

The median follow‐up duration was 8.6 months (95% CI, 7.1–12.9). The best responses observed with therapy were CR in 4/31 (12.9%), PR in 12/31 (38.7%), SD in 5/31 (16.1%), and PD in 7/31 (22.6%) patients. Treatment outcomes by nivolumab dose are shown in Supplementary Table 2. Median duration of response was 4 months (range, 0.8–8.2).

Among all included patients (31), the ORR was 51.6% (95% CI, 33.1%–69.8%) and the DCR was 67.7% (95% CI, 48.6%–83.3%). However, among the evaluable patients (28), the ORR was 57.1% (95% CI, 37.2%–75.5%) and the DCR was 75% (95% CI, 55.1%–89.3%).

The PFS of the entire cohort was 66.1% (95% CI, 50.9%–80.9%) at 6 months, with a median PFS of 8.8 months (95% CI, 6.6‐not reached), and the median OS was 14.8 months. (95% CI, 11.1‐not reached) (Figure 1).

**FIGURE 1:**
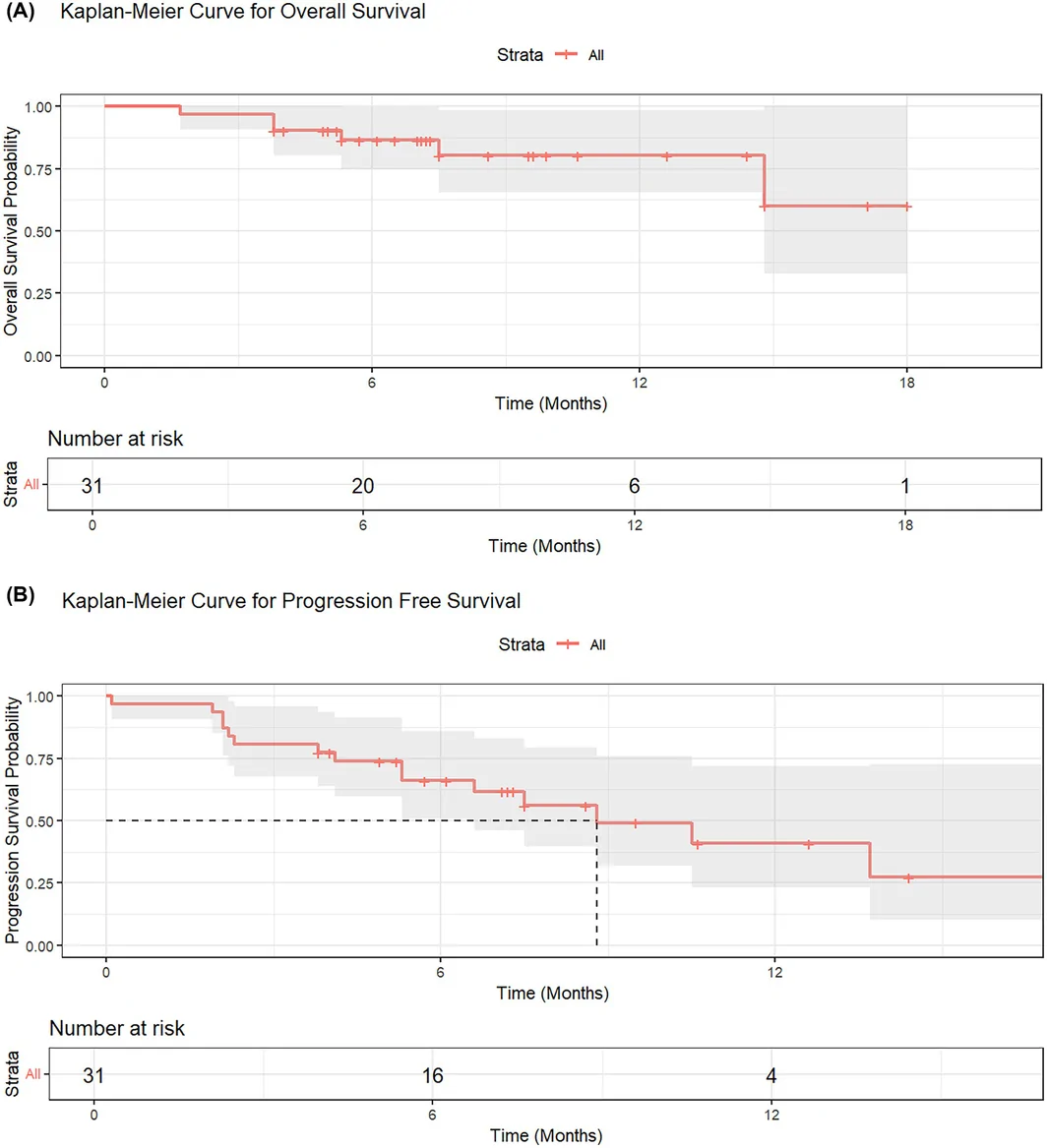
Survival analysis of the patients treated with low‐dose nivolumab. Kaplan–Meier curves (A) overall survival (OS) and (B) progression‐free survival (PFS).

The response of cancer patients to low‐dose nivolumab with respect to treatment duration is shown in the swimmer plot in Figure 2. At the last follow‐up, 3/31 (9.7%) patients had a complete response, 9/31 (29.0%) patients had a partial response, and the disease was stable in 4/31 (12.9%) patients, while there was progression in 6/31 (19.4%) patients, and 6/31 (19.4%) patients died during the study period. CRs, PRs, and stable responses were achieved in an average of 4.7 months, 3.4 months, and 4.3 months of treatment, respectively.

**FIGURE 2:**
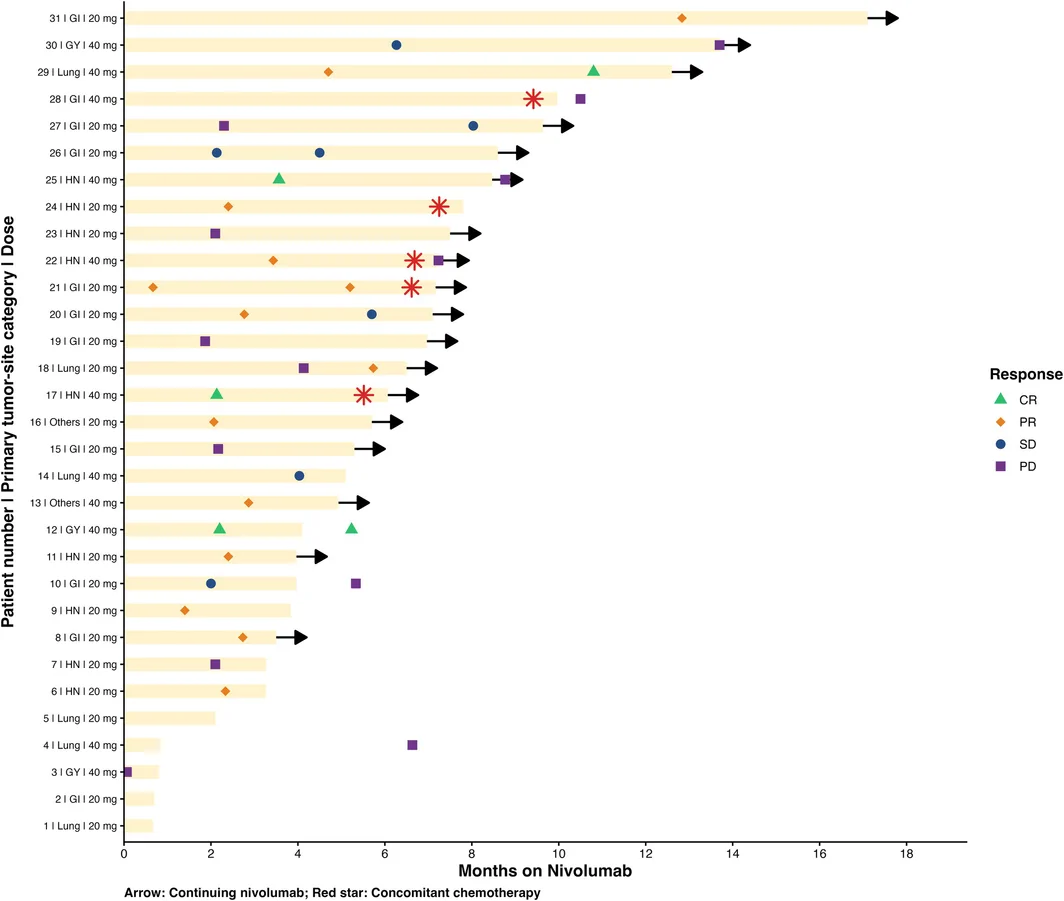
Swimmer plot demonstrating treatment duration and clinical response among 31 patients receiving low‐dose nivolumab. Patient labels indicate the primary tumor site and nivolumab dose. CR, complete response; GI, gastrointestinal cancer; GY, gynecologic cancer; HD, Hodgkin lymphoma; HN, head and neck cancer; PD, progressive disease; PR, partial response; SD, stable disease. Lung and Skin denote lung and skin cancers, respectively. Arrows indicate patients continuing nivolumab at the last follow‐up, and red asterisks indicate patients who received concomitant chemotherapy; their position does not represent the timing of chemotherapy.

Among the patients receiving low‐dose nivolumab, the only adverse events observed were Grade 2 and 3 arthralgia in two patients, diarrhea of Grade 2 in two patients, and effusion of Grade 2 in one patient, as presented in Table 3. No additional Grade 2 or higher adverse events were documented in the available medical records.

**TABLE 3:** Safety assessment.

Adverse event	Grade 2 (*n*)	Grade 3 (*n*)	Grade 4 (*n*)	Total *n* (%)
Arthralgia	1	1	0	2 (6.5)
Diarrhea	2	0	0	2 (6.5)
Pericardial effusion	1	0	0	1 (3.2)

*Note:* Grade 1 adverse events were not systematically captured because of the retrospective study design and are therefore not reported.

## 4 Discussion

Low‐dose nivolumab (20–40 mg every 3 weeks) demonstrated efficacy across various tumors, with an ORR of 51.6%, DCR of 67.7%, median PFS of 8.8 months, and median OS of 14.8 months. These findings align with prior studies showing the efficacy of low‐dose nivolumab in diverse cancer types [5, 10].

Patel et al. observed comparable DCR produced by low (40 mg), per‐weight, and conventional doses of ICIs in various cancers [6]. Likewise, Gandhi et al. achieved a similar ORR with 40 mg and conventional‐dose nivolumab in advanced cancers [11]. Yoo et al. reported no significant difference in clinical response rate, PFS, and OS produced by low (20 and 100 mg) and standard‐dose nivolumab in nonsmall cell lung cancer (NSCLC) [5]. Similarly, Chen et al. demonstrated no statistical difference in DCR of 20 and 100 mg nivolumab in hepatocellular carcinoma [12]. Noronha et al. in a recent Phase III randomized trial achieved significantly longer median OS of 5.88 months versus 4.7 months and a better 1‐year OS rate of 27.3% versus 16.9% between 20 mg nivolumab and standard chemotherapy in various tumors [13]. Patil et al. gained a significant improvement in 1‐year OS with 20 mg nivolumab to standard chemotherapy in head and neck cancer [14].

While CR in 3 and PR in 9 among 31 patients were achieved, 5 patients of our cohort (including 1 with CR and 2 with PR) were also administered chemotherapy along with nivolumab, which introduced heterogeneity in the treatment scheme. However, a combination of traditional treatments, including chemotherapy with immunotherapy, has been reported to enhance the sensitivity of tumors toward immunotherapy [15].

Treatment with low‐dose nivolumab did not raise concerns of toxicity in our patients. Manageable toxicities of Grade 2 or 3 were observed in a few patients. The low rate of toxicity observed in our cohort may be attributed to several factors. The low‐dose nivolumab is better tolerated by patients compared to the conventional dose, as reported by Gandhi et al. [11]. Moreover, the short follow‐up duration and a retrospective design contribute to underreporting of the adverse events.

Financial toxicity was still an issue for our patients, as four patients did not continue treatment, out of whom one patient was in complete remission and one had a partial response.

The low‐dose approach in immunotherapy is an active area of research in different parts of the world, focused on improving access to these innovative drugs. In support of the therapeutic effectiveness of low‐dose immunotherapy, Gandhi et al. have recorded comparable dose‐normalized geometric means of Cmax (0.28 vs. 0.23 μg/mL/mg) and AUC₀₋*ₜ* (0.0014 vs. 0.0011 day/mL) between the conventional (3 mg/kg) and low‐dose nivolumab (40 mg flat) groups, indicating similar systemic exposure per unit dose and demonstrating the pharmacokinetic efficiency of the low‐dose regimen [11]. The evidence of the low‐dose regimen's efficacy prompted Ratain et al. to suggest therapeutic drug monitoring to establish precise doses for individual patients. This could optimize scheduling, potentially reducing dosage or frequency, given nivolumab's long half‐life of 25 days [16].

The formulation of ICIs like nivolumab in liquid multiuse vial form and its physical stability has made low‐dose therapy practically easier to administer, as it has been reported that nivolumab is stable for at least 1 month at 2°C–8°C and 7 days at 40°C in partially used vials [17]. Along with the evidence of low‐dose being equally effective as the recommended dose and its use in clinical practice, the availability of biosimilar products in our market has reduced the total cost of care with ICIs.

Socioeconomic status and cost of therapy are significant barrier to access to immunotherapy, especially in LMICs [18]. Nivolumab generally costs $7/mg in our healthcare setting. Our cohort of low‐dose nivolumab (20–40 mg every 3 weeks) achieved substantial cost savings of 80%–90% compared to standard doses (240 mg every 2 weeks or 480 mg every 4 weeks), enhancing access (Supplementary Table 1). Along with lower dosage, by leveraging vial sharing and adopting a biosimilar nivolumab, the treatment cost of nivolumab can be reduced, improving its access.

The present study has several limitations that must be acknowledged. The retrospective, single‐center study design introduces potential selection bias. Short follow‐up duration and small sample size reduce statistical power. Inconsistent documentation of Grade 1 adverse events may have resulted in underreporting of treatment‐related toxicity. Therefore, these findings should be interpreted cautiously due to potentially confounding factors like the retrospective design, heterogeneous population, and lack of a control group.

## 5 Conclusion

The retrospective study suggests that low‐dose nivolumab shows efficacy in clinical settings, potentially offering cost‐effective treatment options, particularly in resource‐limited settings. However, these findings are hypothesis‐generating and further research is warranted to confirm efficacy, explore optimal dosing, and the safety of low‐dose nivolumab. If validated, this could suggest potential for improving cancer management in resource‐constrained settings.

## Author Contributions

**Subhas Pandit:** conceptualization, writing – original draft, writing – review and editing, methodology. **Simit Sapkota:** methodology, writing – review and editing, conceptualization. **Abish Adhikari:** methodology. **Jeebana Bhandari:** formal analysis, writing – original draft, data curation, conceptualization. **Sunil Shrestha:** writing – review and editing. **Rajani Jha:** writing – review and editing. **Gerim Prasai:** writing – review and editing. **Anjani Kr. Jha:** supervision.

## Funding

The authors have nothing to report.

## Conflicts of Interest

The authors declare no conflicts of interest.

## Supporting information


**Supplementary Table 1.** Local acquisition costs for standard dose versus low‐dose nivolumab as of 2026/2/15.
**Supplementary Table 2**. Treatment outcome of 40 mg versus 20 mg low‐dose nivolumab.

## Data Availability

The data that support the findings of this study are available on request from the corresponding author. The data are not publicly available due to privacy or ethical restrictions.
