# Supplementary material for: Low‐Dose Nivolumab in Various Cancers: A Real‐World Exploratory Analysis in Resource‐Constrained Settings
Source: Cancer Rep (Hoboken). 2026 Oct 19;9(10):e70713. doi: 10.1002/cnr2.70713 (PMC13680289; doi:10.1002/cnr2.70713)
Supplement: Supplementary file 1 [file CNR2-9-e70713-s001.docx]

**Supplementary Table 1:** Local acquisition costs for standard dose vs. low-dose nivolumab as of 2026/2/15

| Nivolumab Dose | 240 mg | 40 mg | 20 mg |
| --- | --- | --- | --- |
| Cost (USD) | 1,680 | 280 | 140 |

**Supplementary Table 2:** Treatment outcome of 40 mg vs 20 mg low-dose nivolumab

| **Response** | **20 mg (N=20)** | **40 mg (N=11)** |
| --- | --- | --- |
| CR | 0 | 4 |
| PR | 8 | 1 |
| SD | 3 | 1 |
| PD | 4 | 2 |
| **Line of Therapy** | | |
| First | 6 | 2 |
| Second | 11 | 5 |
| Third | 2 | 3 |
| Fourth | 1 | 1 |
|  | 4 deaths, response not evaluated in 1 | 2 deaths, response not evaluated in 1 |
